# Experiences and challenges for EQA providers in assessing the commutability of control materials in accuracy-based EQA programs

**DOI:** 10.3389/fmed.2024.1416642

**Published:** 2024-08-01

**Authors:** Laura Vierbaum, Patricia Kaiser, Michael Spannagl, Folker Wenzel, Mario Thevis, Ingo Schellenberg

**Affiliations:** ^1^INSTAND e.V., Society for Promoting Quality Assurance in Medical Laboratories, Duesseldorf, Germany; ^2^Institute of Laboratory Medicine, Ludwig-Maximilians-University Munich, Munich, Germany; ^3^Faculty of Medical and Life Sciences, Furtwangen University, Villingen-Schwenningen, Germany; ^4^Institute of Biochemistry/Center for Preventive Doping Research, German Sport University Cologne, Cologne, Germany; ^5^Institute of Bioanalytical Sciences (IBAS), Center of Life Sciences, Anhalt University of Applied Sciences, Bernburg, Germany

**Keywords:** external quality assessment (EQA), proficiency testing (PT), commutability, reference measurement procedures, trueness control, traceability, measurement accuracy

## Introduction

External quality assessment (EQA) programs in medical laboratory diagnostics are necessary for gaining insight into the analytical performance of a large number of analytes across laboratories. The evaluation of EQA results is either based on consensus values of individual measurement procedures (MPs) or is accuracy-based using a reference measurement value (RMV) as target value for all MPs if a reference MP (RMP) has been established for the analyte. RMPs achieve the highest possible analytical accuracy as they are, ideally, matrix-independent and are comparatively insensitive to interfering substances. In contrast, MPs of a lower metrological order, as are commonly used for routine diagnostics in medical laboratories, can be affected to varying degrees by the sample matrix and components. That is depending on the analytical performance of the respective MP.

Thus, if an RMV can be assigned as a target value to the EQA material (EQAM), EQA programs can serve as measurement trueness controls (1). However, this presupposes that the EQAMs are suitable for measurement with all MPs (2). Since control materials (CMs) are usually processed, their exchangeability with patient samples, also known as commutability (3–5), should be aimed for and is increasingly being called for in professional circles. Due to the required use of pathological analyte concentrations, and the need for samples for up to several hundred participating laboratories, the use of native single donor samples in EQA schemes is not feasible from an ethical point of view, and pooling and spiking of CMs is common. In addition, CMs are commonly stabilized by means of stabilizing additives or lyophilization. While the RMPs used to assign target values ideally remain unaffected by the processed nature of EQAMs, individual routine MPs might be affected, resulting in artificial shifts in MP-specific bias. MP-specific effects on the analysis due to the processing of the material tend to be more critical for analytes with a more complex or possibly tertiary structure, such as proteins (6, 7), than for lower molecular mass analytes with a simpler structure, such as urea. Thus, if observed bias are in part due to a lack of commutability of the EQAM, an EQA evaluation is only possible within the MP collectives based on the consensus value and not on the level of accuracy.

Hence, it is clear that the use of commutable CMs is beneficial to improve quality assurance in medical laboratories. However, the investigation of commutability poses enormous challenges for EQA providers worldwide and requires elaborate practice-oriented concepts.

## Models for commutability assessments

Commutability assessment approaches were originally developed in the field of clinical chemistry, where RMPs are established for some analytes and higher order reference standards exist. However, possible influences of matrix effects on the measurement results for CMs are relevant in all disciplines, e.g., hematology, immunology or virology, and the statistical models for assessing the commutability of CMs described in guidelines can be transferred to analytes in other fields to the extent possible.

It is recommended measuring the analyte in at least 30 native patient samples and measuring CMs using as many MPs on the market as possible. The measurement results of two MPs in any combination can then be correlated, recommended as Deming regression (3), or as a difference in bias plot, also known as a Bland-Altman plot (4, 5). If an RMP is available for the analyte in question, the measurement results of all MPs can only be correlated with the RMVs. A range is then defined based on the correlations of the patient sample values and used as a commutability criterion for the CMs.

The relevance of such models for assessing commutability of processed CMs is beyond question, but the challenges involved in the practical implementation of such theoretical models are virtually impossible for EQA providers to realize. Consequently, limitations prevail that necessitate consideration and awareness as discussed in the following.

## Recruitment of donors for commutability assessment and limitations

To obtain correlations of the measured values that are representative of patient samples, these should cover the broadest possible concentration range of the respective analytes. Recruiting 30 donor materials takes great effort and pushes the limits of what is feasible. Firstly, the targeted selection of suitable donors requires a pre-characterization of numerous patients. Secondly, and most critically, the donation of samples in the range of pathological values is ethically debatable. But MPs can deliver conspicuous results, especially in high or low concentration ranges (8, 9), so that including such patient samples in commutability assessments might be crucial. To avoid freezing patient samples, which can cause, for example, changes in protein structures, all donations for commutability assessment must be collected and processed on the same day and measured immediately by as many MPs as possible. But some analytes have a high biological variability, so a pre-characterization of patients at a certain time would not assist in patient selection to cover the desirable broad concentration range with the fresh specimens. For example, blood parameters, like glucose or electrolytes, fluctuate substantially depending on food intake or fluid balance (10).

## Definition of acceptability criteria and limitations

Ultimately, it is questionable whether even 30 patient samples are sufficient to represent the diverse patient profile and to make a fundamental statement on the commutability of CMs on this basis. Moreover, these models for commutability assessment do not take into account the fact that patient samples may also contain interfering substances for individual MPs, especially those from ill or medicated patients. The fundamental question that arises when we assess commutability is what are suitable criteria for selecting reference patient samples.

While the criteria for commutability of CMs might be narrowly defined based on exemplary patient samples, possible MP weaknesses may be missed. The IFCC Working Group states that MPs with inadequate precision are not suitable for assessing commutability with the Bland-Altman plot, as this might impact the assessment (4). When using the Bland-Altman plot with medically-diagnostically defined criteria, patient samples might also appear to be non-commutable if inadequately precise MPs are included in the assessment. However, when assessing a material, where should the line be drawn between what is considered to be adequate and inadequate in terms of an MP's precision? The line is blurred between whether measurement differences are caused by inadequate precision or material effects.

The assessment of commutability of CMs, of course, depends largely on the strictness of the criteria. The commutability criteria in the Bland-Altman plot can be defined and subjectively justified in different ways. Tight criteria can lead to inconclusive results as the values of the CMs including the measurement uncertainties must fit the criteria (6). Due to this leeway in defining the criteria for the Bland-Altman plot on the one hand, and, the statistically defined criteria for the Deming regression on the other, it is not surprising that an application of both models for one and the same data set yields different results (11).

## The role of measurement performance of MPs

The evaluation of EQA results based on RMVs can reveal a bias in an MP and insufficient standardization of a diagnostic test system. Biased results can indicate an MP's lack of accuracy, yet bias can also be caused by an EQAM's lack of commutability. However, it is hard to identify the contributions of these two parties to an observed measurement bias. It should be noted that the analytical selectivity of an MP to interferences also determines whether it is affected by matrix effects or sample additives. Analyses of data from past EQA surveys show that MPs of the market-leading manufacturers deliver measurements with varying degrees of robustness and accuracy. However, individual MPs manage to reliably deliver very precise and some also very accurate results (12, 13), even when measurements are conducted on EQAMs of a processed nature.

Lack of specificity or low robustness of certain MPs have also been identified in studies with clinical samples and are the core reason behind unreliable laboratory diagnostics (14, 15). Measurement results should be reliable, especially for “conspicuous” patient samples, e.g., for samples from patients under the influence of medication, where undesirable disturbances in the analysis can occur more frequently (16).

The measurement of creatinine is an example of a clearly divided distribution of INSTAND EQA results depending on whether kinetic or enzymatic methods were used for the analysis.^1^ The results from interlaboratory testing, classified as a category 1 EQA scheme, on samples assessed as commutable (17) show that serum creatinine measurements were more accurate using enzymatic methods than kinetic ones (18–20). The Jaffe method reagent is known to be sensitive toward reacting with several interfering components in serum such as glucose, bilirubin, or hemoglobin, which is consequently critical for measurements on icteric or hemolytic samples. Thus, a lack of specificity in the kinetic creatinine measurement produces overestimated values, especially in the case of lower creatinine concentrations (21, 22).

For other measurands in clinical chemistry, there are no means of metrological traceability for the values. For example, there are no high-order MPs or primary standards for procalcitonin measurement (23). In such circumstances, a high variability in EQA results is not surprising and an evaluation of the analytical performance of a laboratory can only be made based on consensus values.

## Challenges for EQA providers

Commutability assessment studies are quite feasible for certified reference materials that are usually produced on a large scale. However, the situation is different for EQA providers who offer EQA schemes for many analytes in laboratory medicine and who manage a high throughput of batches per scheme and year. Consequently, an enormous number of studies would be necessary to investigate the commutability of the high number of different EQAM batches. With the requirement for commutable CMs, EQA providers are challenged by what is feasible and financially viable. The high number of patient samples required for material assessment, even in pathological concentration ranges, appears very paradoxical and practically impossible to implement when one considers that processed materials are deliberately used in EQA schemes, particularly for ethical reasons. Severely ill patients in the areas of hematology, immunohematology, and oncology, including those undergoing therapy, cannot have large quantities of blood taken so that EQA schemes can be conducted with several hundred participating laboratories or numerous commutability studies can be performed to characterize the EQAMs. Also for ethical reasons, samples from patients with rare diseases cannot be included in such surveys. The availability of patient materials for studies is also severely limited if the collection of the material is associated with increased medical intervention, as in the case of cerebrospinal fluid. The applicability of the models for assessing commutability is thus severely limited to clinical chemistry parameters.

Commutability assessments of EQAMs is far from feasible for MPs that are less prevalent on the market, and especially for in-house products. EQA providers can only include the market-leading MPs in commutability studies in cooperation with representative measurement centers.

In order to significantly reduce the effort for EQA providers to provide commutability studies for all EQAM batches, it is sometimes assumed that the results of a study can also be applied to identically produced CMs. However, it is known that lot-to-lot variability can occur even with identically produced sample or assay batches (24). Occasional effects in EQAM batches are represented by a conspicuous and unusual scattering in the MP-specific value distribution, e.g., as observed in 2022 in an INSTAND EQA scheme for the quantitation of 17β-estradiol (12). Samples with such conspicuous results must be excluded from the EQA evaluation.

Overall, the effort needed for the commutability studies is a challenge that cannot currently be overcome in practice. EQA providers can only check in bullet points and to a limited extent the commutability of the EQAMs. Due to limitations in the implementation of commutability studies, which include the lack of availability of patient samples with pathological concentrations, a focus on market-leading methods, and varying criteria definitions, the information obtained from these studies should be balanced against the enormous effort that they entail. Obtaining a broader and more comprehensive picture is certainly scientifically desirable, however, the inevitable limitations of the assessments, costs, and EQA fees become incompatible with client needs. Hence, it appears that the only way to achieve progress regarding the challenge of assessing the commutability of EQAMs in a practical manner necessitates the cooperation of sample manufacturers, national metrology institutes, and IVD manufacturers and cannot be handled by EQA providers alone.

Ultimately it is the aim of EQA providers to offer the most suitable CMs possible to support reliable measurement results in medical laboratories. Comparative studies with patient materials on a smaller scale and empirical values from the literature can provide valuable indications whether significant sample effects are expected for an EQAM.

## Relevance of the exact study design

The widespread dissemination of the theoretical commutability assessment models in medical and scientific communities has led to increased demand for commutable CMs. In reaction to this, CMs are increasingly being declared commutable without providing more detailed information on the study design.

Commutability is not a property that can be attributed exclusively to the material but must be regarded as being a direct result of the exact study design (25). At the very least, information needs to be provided about the MPs and the defined assessment criterion involved in order to gain an accurate picture of a material's commutability. The complexity of commutability assessments is often not considered in its entirety in professional circles and by CM end users. In light of the fact that the implementation of commutability studies necessitates major and minor limitations, uncommented statements on the commutability of CMs should always be interpreted critically.

## Conclusion

The aim of EQA providers is to promote quality assurance in medical laboratories. Thus, it is in their interest to provide EQAMs that are as suitable as possible for the purpose of the EQA. However, the models for commutability assessment of CMs are only theoretical models that reach their limits in practice in terms of their practicability. Assessing all EQAM batches is simply not manageable and sporadic assessments come up against several limitations. In particular, the availability of patient samples with pathological concentrations is critical and a focus on market-leading MPs is necessary. Statements on commutability must necessarily be interpreted within the context of the entire study design. Ultimately, the information gained from these assessments should be balanced against the enormous effort involved, and practice-oriented concepts need to be developed, which would greatly benefit from the cooperation between all parties involved, EQA providers, sample manufacturers, national metrology institutes, and IVD manufactures in internationally active networks.

## Author contributions

LV: Conceptualization, Methodology, Writing – original draft, Writing – review & editing. PK: Supervision, Writing – review & editing, Conceptualization, Methodology. MS: Supervision, Writing – review & editing, Methodology. FW: Writing – review & editing, Supervision. MT: Supervision, Writing – review & editing. IS: Methodology, Supervision, Writing – review & editing, Conceptualization, Project administration.

## Footnotes

^1^INSTAND *RV-Online* [Online]. Available online at: http://rv-online.instandev.local/index.shtml?lang=en.
